# Beneficial impact of epigallocatechingallate on LDL-C through PCSK9/LDLR pathway by blocking HNF1α and activating FoxO3a

**DOI:** 10.1186/s12967-020-02362-4

**Published:** 2020-05-12

**Authors:** Chuan-Jue Cui, Jing-Lu Jin, Lin-Na Guo, Jing Sun, Na-Qiong Wu, Yuan-Lin Guo, Geng Liu, Qian Dong, Jian-Jun Li

**Affiliations:** Faculty of Division of Dyslipidemia, State Key Laboratory of Cardiovascular Disease, Fu Wai Hospital, National Center for Cardiovascular Diseases, Chinese Academy of Medical Sciences, Peking Union Medical College, Beijing, 100037 China

**Keywords:** Epigallocatechingallate, Proprotein convertase subtilisin/kexin type 9, Low density lipoprotein receptor, Low density lipoprotein cholesterol, Hepatocyte nuclear factor-1α, Forkhead box class O 3a

## Abstract

**Background:**

Green tea drinking has been proven to lower lipid and exert cardiovascular protection, while the potential mechanism has not been fully determined. This study was to investigate whether the beneficial impact of epigallocatechingallate (EGCG), a type of catechin in green tea on lipids is associated with proprotein convertase subtilisin/kexin type 9 (PCSK9) pathways.

**Methods:**

We studied the effects and underlying molecular mechanism of EGCG or green tea on regulating cholesterol from human, animal and in vitro.

**Results:**

In the age- and gender-matched case control observation, we found that individuals with frequent tea consumption (n = 224) had the lower plasma PCSK9 and low density lipoprotein cholesterol (LDL-C) levels compared with ones without tea consumption (n = 224, p < 0.05). In the high fat diet (HFD) fed rats, EGCG administration significantly lowered circulating PCSK9 concentration and liver PCSK9 expression, along with up-regulated LDL receptor (LDLR) expression but decreased level of LDL-C. In hepatic cell study, similar results were obtained regarding the impact of EGCG on LDLR and PCSK9 expression. The assay transposase-accessible chromatic with high-throughput sequencing (ATAC-seq) and subsequent results suggested that two transcription factors, hepatocyte nuclear factor-1α (HNF-1α) and forkhead box class O (FoxO) 3a involved in inhibitory action of EGCG on PCSK9 expression.

**Conclusions:**

The present study demonstrates that EGCG suppresses PCSK9 production by promoting nuclear FoxO3a, and reducing nuclear HNF1α, resulting in up-regulated LDLR expression and LDL uptake in hepatocytes. Thereby inhibiting liver and circulating PCSK9 levels, and ultimately lowering LDL-C levels.

## Background

Cardiovascular disease (CVD) is one of the major worldwide health threaten with severe morbidity and mortality, and dyslipidemia is a critical risk factor for CVD [1]. Low-density lipoprotein receptor (LDLR) in liver plays an important role in maintenance plasma cholesterol levels by regulation of circulating LDL clearance. An elevation of the cholesterol (LDL-C) level in the plasma leads to hypercholesterolemia. The LDL-LDLR complex is internalized through LDLR-mediated endocytosis, followed by lysosomal degradation of LDL, but the LDLR is recycled on the cell surface [2].

Proprotein convertase subtilisin kexin 9 (PCSK9) was discovered in 2003 [3]. It is a member of the mammalian proprotein convertase family of subtilisin-like serine endoproteases, and is located mostly in the liver, much less in the small intestine and kidney, and expressed instantaneously in the developing central nervous system [4]. More and more evidences indicated that PCSK9 plays an important role in lipid metabolism [5–7]. It can regulate cholesterol metabolism by reducing hepatic LDLR. PCSK9 can accelerate the degradation of liver LDLR and prevent its recycling to the cell surface, which reduces the removal of lipid from the liver and increases the levels of lipid, thus promoting atherosclerosis [8]. Genetic studies showed that humans with nonsense or missense mutations in PCSK9, causing loss-of-function variants of PCSK9, have significantly lower levels of plasma LDL-C than normal and are protected from atherosclerotic cardiovascular diseases [9–11]. It can be seen that inhibitions of PCSK9 may reduce the level of circulating LDL-C by activating its uptake [12, 13].

Green tea has previously been demonstrated to be a healthy beverage for cardiovascular system [14]. It is a tradition Asian beverage made from the dried leaves of tea trees and contains rich polyphenol catechins, which can be divided into four categories including epicatechin (EC), epicatechingallate (ECG), epigallocatechin (EGC), and epigallocatechingallate (EGCG) [15]. Among these catechins, EGCG is the highest, accounting for 58% of the total catechins in green tea [16]. In fact, epidemiological and animal studies have identified that intake of green tea may play an important role in lipid metabolism [17–19]. However, there are a few studies concerning the underlying mechanisms of EGCG on the effect of lowering lipid [20].

In this study, we illustrate that EGCG can reduce hepatic PCSK9 expression, which were associated with increased levels of liver LDLR and subsequent reduction of plasma circulating PCSK9 concentration and cholesterol levels. This study provides scientific evidences on green tea as a natural PCSK9 inhibitor regulates cholesterol.

## Materials and methods

### Human study and population

The present study was approved by the Ethics Committee of the Fu Wai Hospital and informed consent was signed by all participants signed. We consecutively enrolled 763 patients who had not administered any second-level prevention drugs including statins and scheduled for physical examination from January 2015 to July 2016. Information of tea consumption was obtained by direct interviewing. The definition for the frequent tea consumption drinkers was those of patients drinking at least one cup (250–300 mL) of green tea daily, and non-drinker as patients drinking no green tea at all. Each patient who regularly green tea drinker was matched to 1 randomly selected controls on age and gender. Finally, 224 cases and 224 controls were included.

### Examinations for human samples

The fasting blood samples were collected from all patients before the diagnostic procedures and medication treatment. As previously described [21], after centrifugation at 3000 rpm for 10 min at 4 °C, the plasma were collected and stored at − 80 °C. The LDL-C concentrations were measured using an enzymatic assay by automatic biochemistry analyzer (Hitachi 7150, Tokyo, Japan). In consistent with our previous studies [21], the plasma PCSK9 concentrations were measured by a high-sensitivity, quantitative sandwich enzyme immunoassay (Quantikine ELISA, R & D Systems Europe Ltd) with the lowest limit of detection of 0.096 ng/mL.

### Animals and treatment

Eight-week-old Sprague-Dawly (SD) male rats, weighing 180 ± 20 g at the beginning of the study, were provided by the Beijing vital river laboratory animal technology Co., Ltd. (Beijing, China). All experimental procedures were approved by the Ethics committee for animal care and research at Beijing laboratory animal research center (Beijng, China; approval BLARC-2017-H-007). The rats were individually housed in plastic cages and acclimatized to the standardized environment (a 12-h-light–dark cycle, a temperature of 21 ± 1 °C,a relative humidity of 50 to 70% and 20% air changes per hour) for 1 week. Then the rats were randomized and fed with normal chow diet (ND), and high fat diet (HFD) which is composed of 1% cholesterol, 68.6% basal diet, 10% lard, 10% fructose, 10% of egg yolk powder, 0.2% bile salts, and 0.2% methylthiouracil [22]. After completion of 4 weeks of HFD feeding, HFD rats were divided into the following groups and administered by intragastric gavage (i.g.) at different doses of EGCG (purify ≥ 95%,Meilun Biological technology Co., Ltd., Dalian, China) for another 4 weeks: HFD control: HFD feeding and treated with normal saline (i.g. normal saline); EGCG Low: HFD feeding and treated with low- dose EGCG (i.g. 50 mg/kg/d), EGCG High: HFD feeding and treated with high-dose EGCG (i.g. 200 mg/kg/d). At the end of the treatment, all rats were fasted overnight and sacrificed to collect blood and livers samples for analysis.

### Cell culture and treatment

The HepG2 cell line and Huh7 cell line were obtained from Cell Resource Center, IBMS, CAMS/PUMC (Beijing, China). Cells were maintained in DMEM (Hyclone, Logan, UT, USA) with 10% fetal bovine serum (FBS) (Gibico, NY, USA) and 1% NEAA (Life technologies, California, Carlsbad, USA) at 37 °C in an incubator containing humidified air with 5% (v/v) CO2, and passaged at 90% confluency with 0.25% (w/v) trypsin-EDTA (Life technologies, California, Carlsbad, USA). The cells were changed to Minimum Essential Medium (MEM) supplemented with 5% lipoprotein-deficient serum (LPDS) (Sigma, St. Louis., MO, USA) overnight. Cells were then treated with EGCG (purity ≥ 95%,Sigma, St. Louis., MO, USA) for an additional 24 h.

### MTS toxicity analysis

Cell viability was determined by a Cell Titer 96 Aqueous ONE Solution Reagent (MTS) colorimetric assay (Promega, Madison, WI, USA) [23]. Briefly, cells were treated with the EGCG (25–200 μM) for 24 h followed by incubation with 20 μL of MTS solution each well of 96 well- plate at 37 °C for 2 h. Absorbance of each well was read at 490 nm. Repeat the experiment three times.

### Real-time polymerase chain reaction (PCR) assay in cells

To detect the effect of EGCG on the messenger ribonucleic acid (mRNA) level of PCSK9 and LDLR in HepG2 cells, we performed Real-time PCR. Total RNA was extracted and reverse-transcribed (RT) from control and EGCG treatment group in HepG2 cells. Quantitation of PCSK9 transcript levels was performed by amplification of complementary deoxyribonucleic acid (cDNA) prepared from the isolated RNA with ABI 7500 (Applied Biosystems Inc., Foster City, CA, USA), using the SYBR green PCR master mix (Applied Biosystems Inc., Foster City, CA, USA) and primers specific for PCSK9 (forward, 5′-ATC CAC GCT TCC TGC TGC -3′; reverse, 5′-CAC GGT CAC CTG CTC CTG-3′), LDLR (forward,5′-AGG AGA CGT GCT TGT CTG TC-3′; reverse, 5′-CTG AGC CGT TGT CGC AGT-3′) and glyceraldehydes-3-phosphate dehydrogenase (GAPDH) as an internal control (forward, 5′-GCA AAT TCC ATG GCA CCG T-3′; reverse, 5′-TCG CCC CAC TTG ATT TTG G -3′) (Takara, Dalian, Liaoning, China). The total volume is 20 μL for each sample, and the program as following: 50 °C for 3 min, 95 °C denaturation for 10 min, PCR consisted of 40 cycles, including 15 s at 95 °C, 1 min at 60 °C, followed by a 7 min extension at 72 °C. Differences in threshold cycles number were used to quantify the relative amount of PCR target contained within each tube. Relative mRNA species expression was quantitated and expressed as transcript accumulation index (TAI = 2^− (△△CT)^), calculated using the comparative CT method. All values were normalized to the constitutive expression of the housekeeping gene, GAPDH. Repeat the experiment three times.

### Western blots assay in cells and livers

To study the molecular mechanism of EGCG on regulating lipid, the expressions of nuclear protein or total protein in cells and rat livers were detected by western blots assay. Hepatocytes and rat liver tissue samples were homogenized on ice in lysis buffer containing protease and phosphatase inhibitors (Beyotime, Shanghai, China). The homogenate was then centrifuged at 12,000*g* for 15 min and the supernatant was collected. For isolation of nuclear extracts, the cells were harvested using the Nuclear Extraction Kit (Millipore, Billerica, MA, USA) according to the manufacturer’s instructions. Protein concentration was determined using a BCA protein assay kit (Beijing Kangwei century biotechnology Co., Ltd., Beijing, China). Subsequently, 50 μg of protein from individual samples was separated by precast NuPAGE Novex 4–12% (w/v) Bis–Tris gels (Life technologies, Carlsbad, CA, USA) and then transferred onto nitrocellulose membrane using the iBlot™ dry blotting system (Life technologies, Carlsbad, CA, USA) as described by the manufacturer. Membranes were stained with Ponceau S and blocked in TBST buffer (20 mM Tris, pH 7.5, 150 mM NaCl, 0.1% of tween 20) containing 5% non-fat dry milk for 1 h at room temperature.

The blots were reacted with primary antibodies overnight at 4 °C, then with a secondary antibody conjugated with horseradish peroxidase (HRP) for 2 h at room temperature. Blots were developed using chemoluminescence (Thermo Fisher Scientific, Waltham, MA, USA) on FluorChem M image system. Repeat the experiment three times.

### DiI-LDL uptake in cells

The amount of LDL absorbed by cells depends on the number of receptors on the cell membrane. To study uptake of LDL in hepatic cells, we carried out the DiI-LDL uptake assay. Briefly, the cells were plated in 24 -well plates and incubated in DMEM medium with 5% LPDS and 20 μg/mL of DiI-LDL (Life technologies, California, Carlsbad, USA) for 24 h at 37 °C in the dark. For the fluorescence microscopy, the cells were fixed in the presence of a 4% paraformaldehyde, and the nuclei were subsequently stained with Hoechst dye. Then the cells were examined with fluorescence microcopy (DMI-4000B, Leica, Männedorf, Switzerland). For the fluorescence quantification, the 10^5^ of cells were scraped and moved into the black 96 -well plate, then 400 μL of isopropanol was added into each well, and 200 μL aliquots were used for the analysis with an infinite M200PRO microplate reader (Tecan, Männedorf, Switzerland, excitation/emission at 530/580 nm). 0.5 mol/L of NaOH was used to lyse the remaining cells and aliquots of 10 μL for protein concentration assay. The ratio of DiI-LDL/protein concentration was used to normalize DiI-LDL uptake value to the cell protein.

### PCSK9 ELISA in rat experiment

To examined the effect of EGCG treatment on the levels of rat plasma PCSK9. Isolated plasma from rat blood samples and determined the levels of PCSK9 using a rat proprotein convertase9/PCSK9 ELISA kit (CycLex Co., Nagano, Japan) and following the manufacturer instructions.

### Determination of circulating LDL-C in rat study

Rats were fasted for 12 h prior to collect the blood samples for analysis. Each blood sample was harvested into a centrifuge tube and allowed to obtain the serum that was separated by centrifugation. The LDL-C concentration was measured by using the commercial kits in 96-well enzyme immunoassay plates.

### Liver histology and immunofluorescence

To observe the location and expression of PCSK9 in the liver from morphological study, we performed the immunofluorescence pathological examination. For histological analysis, liver tissues of all groups were fixed in 4% para-formaldehyde/PBS at 4 °C overnight, embedded in paraffin and sliced at 4 μm thickness. After deparaffinization and hydration, tissue sections were pretreated by heating for 20 min in sodium citrate solution (0.01 M, pH 6.0) in a 95 °C water bath for the antigen retrieval. Prior to blocking with 10% goat serum/TBST for 15 min, tissue sections were treated with 3% H_2_O_2_ for 20 min, then sequentially incubated with rabbit anti-PCSK9 polyclonal antibody (1:100) at 4 °C overnight, followed by incubating with Alexa Fluor^®^ 488-conjugated goat anti-rabbit IgG (1:200, abcam) for 1 h at 37 °C in the dark. After rinsing with PBS, sections were counterstained with DAPI (Zhong ke wan bang technology Co. Ltd, Beijing, China) to show cell nucleus, rinsed three times in PBS, mounted with glycerol/PBS (1:1) and photographed.

### ATAC-seq and analysis in cells

Assay for transposase-accessible chromatic with high-throughput sequencing (ATAC-seq) is a new technology for screening transcription factors [24]. This technology was performed by Annoroad Gene Technology, the protocol as following. Around 50,000 living cells were taken for each library preparation. The cells were lysed in 1 × lysis buffer to get the nuclei, and TruePrep™ DNA Library Prep Kit V2 for Illumina (Vazyme Biotech) was used to construct the transposase -treated libraries. The mass concentration and molar concentration of libraries were detected by Qubit 3.0 Fluorometer and StepOnePlus™ Real-Time PCR system, respectively, and lengths of inserted fragments were detect with Agilent HS 2100 Bioanalyzer. Qualified libraries were sequencing by IlluminaHiSeq X ten platform in pair-end 150 bp style.

Raw data was stored in FASTQ format, including the base sequence and corresponding quality information. Adaptor-polluted or low-quality reads were then filtered out to get the clean data. Clean data was mapped to reference genome by Bowtie2, and visualized by IGV (Integrative Genomics Viewer). Peaks corresponding to the open region in genome were detected by MACS2.

### Small interfering ribonucleic acid (siRNA) transfection in cells

To further explore the mechanism involved in regulating lipid by EGCG, the cells were transfected by siRNA of PCSK9, HNF1α and FoxO3a, respectively. PCSK9 Stealth siRNA duplexes (Life technologies, Carlsbad, CA, USA) targeting sequences: 5′-GAC AUC AUU GGU GCC UCC AGC GAC U-3′ and 5′-AGU CGC UGG AGG CAC CAA UGA UGU C-3′. HNF1α Stealth siRNA duplexes (Life technologies, Carlsbad, CA, USA) targeting sequences: 5′-UCG AUA CCA CUG GCC UCA ATT-3′ and 5′-UUG AGG CCA GUG GUA UCG ATT-3′. FoxO3a Stealth siRNA duplexes (Life technologies, Carlsbad, CA, USA) targeting sequences: 5′-AAC CCU CCA AUG UGU UUC AAC TT-3′ and 5′-GUU GAA ACA CAU UGG AGG GUU TT-3′ [25]. The stealth RNAi negative control Duplex (Life technologies, Carlsbad, CA, USA) were used as a control. RNAi were transfected into hepatic cells using Lipofectamine TM RNAiMAX (Life technologies, Carlsbad, CA, USA) according to manufacturer’s protocol.

### Statistical analysis

The statistical significance was evaluated using Student’s t-test and the violin plots were generated with the R program. Results are presented as the mean ± SEM. All p-values were two-tailed and significant was accepted when p < 0.05.

## Results

### Effects of human circulating PCSK9 and LDL-C levels by drinking green tea

**Fig. 1:**
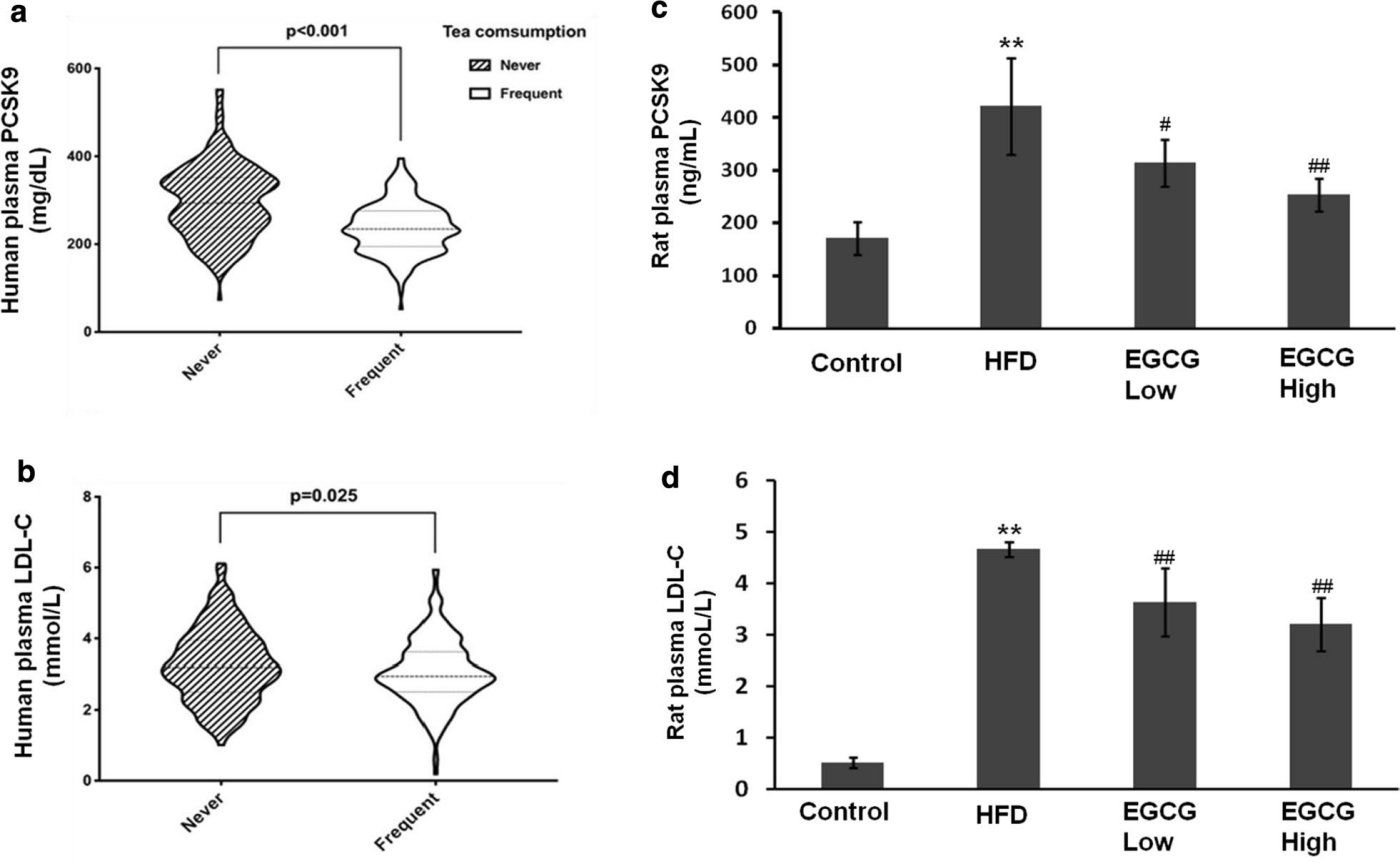
Change in circulating PCSK9 and LDL-C concentrations in human and in HFD rats. Comparisons of PCSK9 (**a**) and LDL-C (**b**) levels between tea drinker and non-tea drinkers by violin plot analysis. The vertical position of each histogram represents the relative levels of PCSK9 or LDL-C. SD rats fed with HFD for 4 weeks and administered with EGCG by i.g. at different doses for another 4 weeks. **c** The levels of plasma PCSK9 were analyzed by ELISA kit. **d** LDL-C lipid levels measured by biochemical kit. **p < 0.01, vs control, ##p < 0.01, versus HFD group (N = 6)

We observed the data of 224 regular tea drinkers and 224 non-tea drinkers control by violin plot analysis. Both of the levels of plasma PCSK9 (236.8 ± 57.7 mg/dL versus 297.4 ± 81.6 mg/dL, p < 0.001) and LDL-C (3.05 ± 0.92 mmol/L versus 3.26 ± 1.02 mmol/L, p = 0.025) were significantly lower in those of regular tea drinkers than non-tea drinkers (p < 0.05) (Fig. 1a, b).

### EGCG reduces plasma PCSK9 and LDL-C in HFD rats

Then, we explored the effects of EGCG on anti-hyperlipidemia activity in HFD rat model. As shown in Fig. 1c, d, higher plasma levels of PCSK9 (421.22 ± 92.1 ng/mL versus 170.85 ± 31.87 ng/mL, p < 0.01) and LDL-C (4.66 ± 0.14 mmol/L versus 0.52 ± 0.10 mmol/L, p < 0.01) in HFD group compared to normal diet group. After treatment with EGCG, it was found that EGCG reduced the levels of plasma PCSK9 (lower dose: 363.80 ± 81.60 ng/mL,p > 0.05; higher dose: 253.38 ± 31.64 ng/mL versus 421.22 ± 92.11 ng/mL, p < 0.01) and LDL-C (lower dose: 3.63 ± 0.66 mmol/L, p < 0.01; higher dose: 3.20 ± 0.52 mmol/L,versus 4.66 ± 0.14 mmol/L, p < 0.01) in HFD rats.

**Fig. 2:**
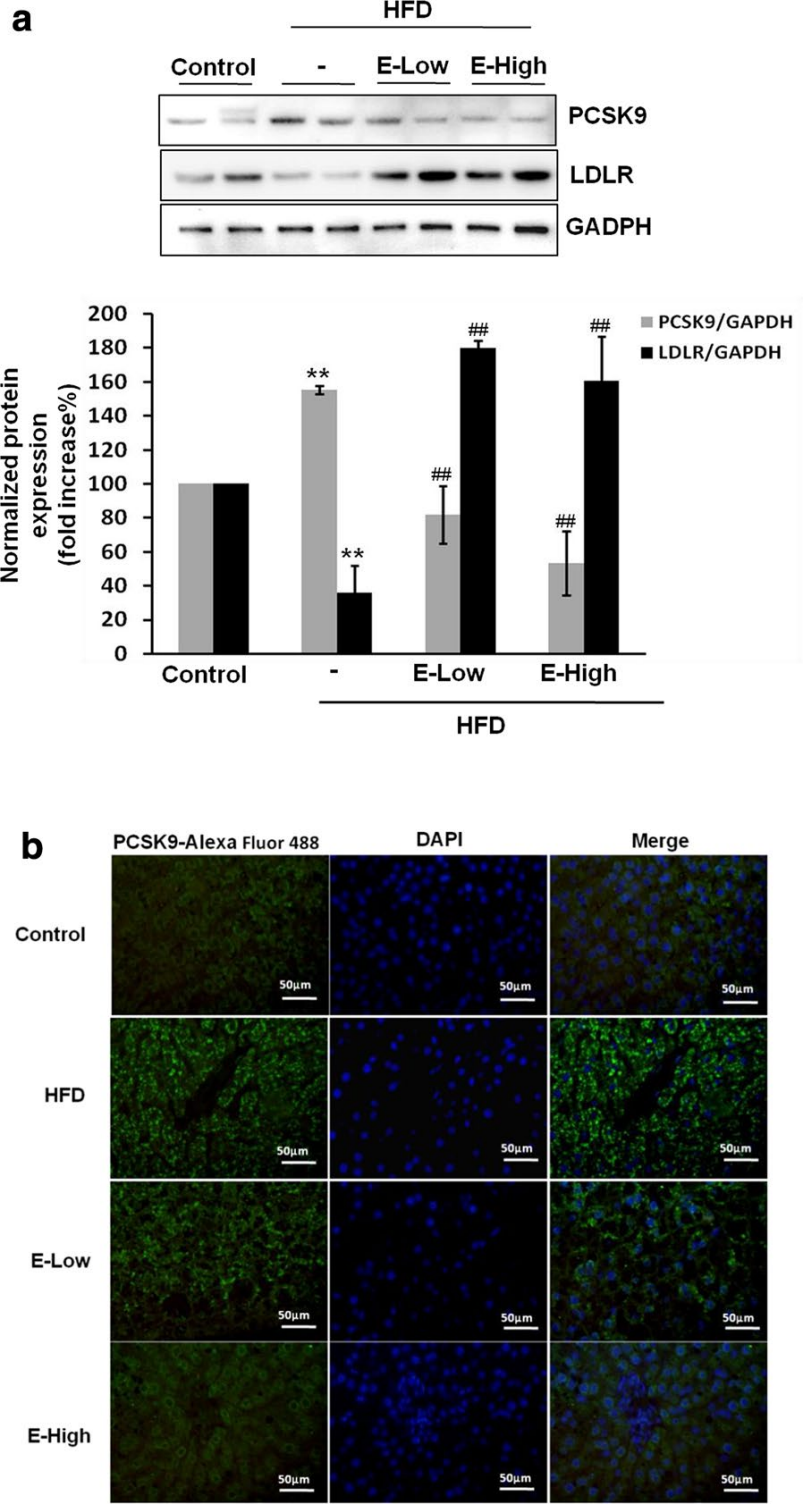
EGCG suppresses PCSK9 expression in hepatic tissue of rats. SD rats fed with HFD for 4 weeks and administered with different doses of EGCG by i.g. for another 4 weeks. **a** The hepatic protein samples were extracted and detected the expression of PCSK9 and LDLR by western blotting and densitomedtric analysis. **b** Hepatic tissue of rats were fixed in 4% paraformaldehyde and paraffin-embedded μm sections subjected to immunofluorescence analysis. Representative images of liver paraffin sections stained for PCSK9 with Alexa Fluor 488 (green) and counter stained with DAPI to show cell nucleus (blue). The photomicrographs were taken by Zeiss AX10 fluorescence microcopy at original magnification × 200. Results are representative of three independent experiments with similar results. **p < 0.01, vs control, ##p < 0.01, versus HFD group (N = 6). E-low: the lower dose of EGCG; E-high: the higher dose of EGCG

Further, we evaluated the effects of EGCG treatment on the expression of PCSK9 and LDLR in liver. The data showed that the expression of PCSK9 was down-regulate about to 52.6 ± 19% and 34.2 ± 17.1% by low dose and high dose of EGCG, respectively, while the expression of LDLR (Fig. 2a) was increased to approximately 5 times in EGCG groups compared to HFD groups. Additionally, it was observed that the levels of PCSK9 in hepatic tissues of HFD rats treated with EGCG were remarkably reduced compared with the group of HFD by immunofluorescence assay (Fig. 2b).

### Effects of EGCG on the viability of cells

**Fig. 3:**
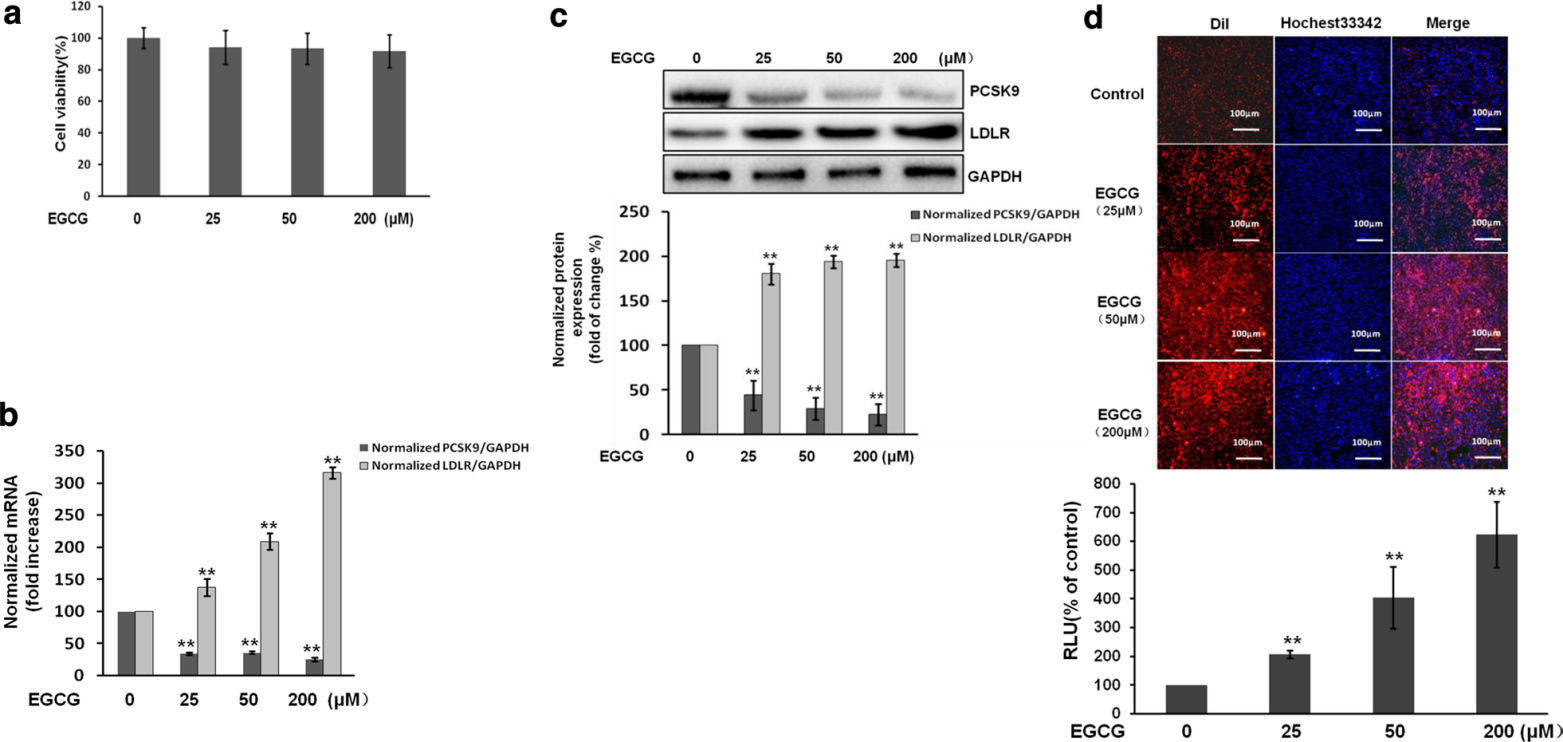
EGCG inhibits the expression of PCSK9, but promotes the LDLR expression and activity in HepG2 cells. The cells were treated with different doses of EGCG (25-200 μM) for 24 h. **a** Cell viability was measured using an MTS assay. **b** The mRNA expression of PCSK9 and LDLR were analyzed by real-time PCR. Relative expression changes are presented as the fold-increase of PCSK9/GADPH and LDLR/GAPDH. **c** The protein expression of PCSK9 and LDLR was analyzed by western blots. **d** Representative fluorescence microscopy images of cell‐associated Dil‐LDL (red), Hoechst‐stained nuclei (blue) and the overlay (upper). Fluorescence of isopropanol‐extracted Dil (520–570 nm, normalized to the cell protein) (lower). Data are presented as mean ± SEM (N = 3). *p < 0.05, **p < 0.01 compared with control

To identify the effects of EGCG on cell viability, HepG2 cells were cultured and treated with different concentration of EGCG (0, 25, 50, 200 μM) for 24 h. It was found that EGCG at concentrations of 25–200 μM had no effect on HepG2 cell viability (Fig. 3a).

### EGCG reduces PCSK9 but promotes LDLR expression and activity in cells

To further investigate whether EGCG regulates the expression of PCSK9 in hepatic cells, we examined the mRNA and protein expression of PCSK9 and LDLR by real-time PCR and western blots in HepG2 cells. The data showed that the mRNA levels of PCSK9 were significantly decreased by approximately 64.5 to 75.4%, and the protein expressions of PCSK9 were also significantly reduced by approximately 55.8 to 77.7%. However, the LDLR mRNA expressions increased by approximately 37.0 to 204.2%, and the protein expressions of LDLR were increased by about 80.6 to 95.9% (Fig. 3b, c).

Moreover, we further examined the effect of EGCG on LDL uptake. As expected, EGCG treatment (0, 25, 50, 200 μmol/L, 24 h) activated LDL uptake in HepG2 cells in a dose-dependent manner (2.1 ± 0.14 times to 6.2 ± 1.1 times) (p < 0.01), compared with the untreated cells (Fig. 3d).

The above results indicated that EGCG increases the levels of the expression of LDLR through the suppression of PCSK9, which leads to activated LDL uptake in hepatic cells.

### Analysis of differential transcription factor in the open chromatin regions using by ATAC-seq

**Fig. 4:**
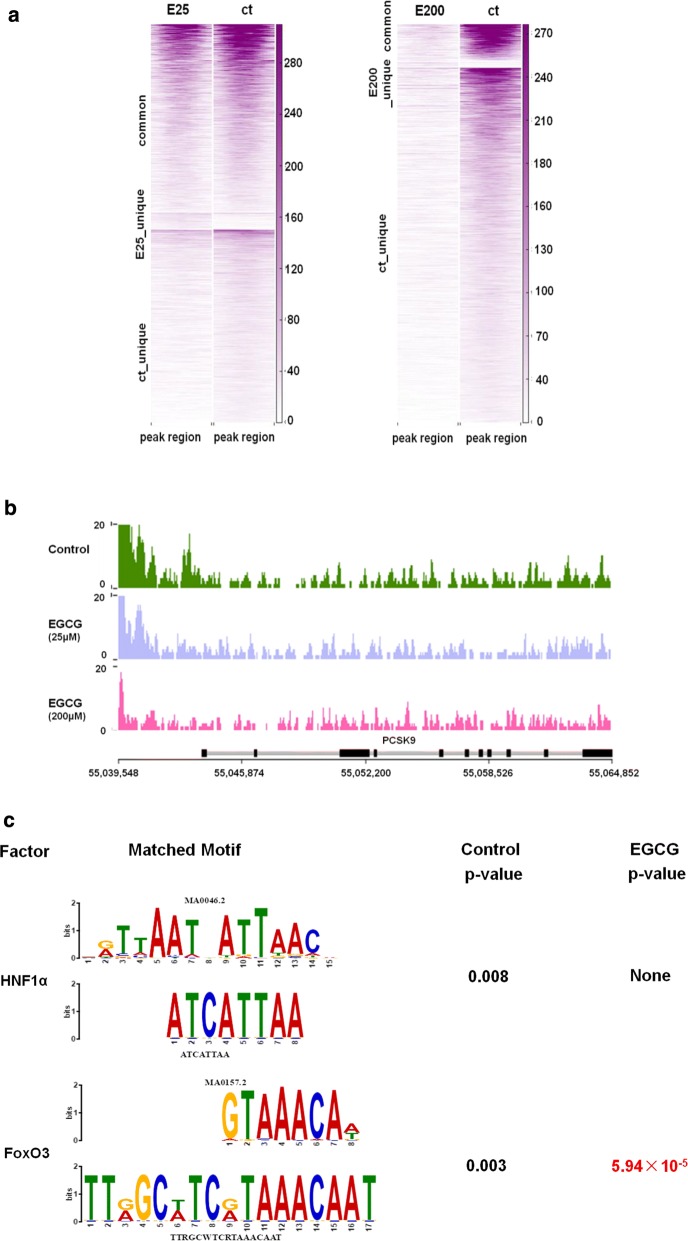
Significantly open chromatin and enriched TF binding motifs using by ATAC-seq analysis in HepG2 cells. The cells were treated with 25 μM of EGCG for 24 h. **a** ATAC-seq analysis of open chromatin heat map. **b** ATAC-seq analysis of open chromatin shows the PCSK9 promoter **c** Significantly enriched TF binding motifs (HNF1α and FoxO3).ct: control group; E25: 25 μM of EGCG; E200: 200 μM of EGCG

One of the main advantages of ATAC-seq is that this technique may indicate potential transcriptional regulator in the disease. Because transcription factor (TF) binds to homologous DNA sequences, known as motifs, often obligates nucleosome eviction and creation of an accessible DNA site, integration of known TF motifs with DNA accessibility data from ATAC-seq can predict the whole genome regulatory network. About 50,000 living cells were performed for the ATAC-seq and obtained statistically significant motifs that the differential transcription in the open chromatin regions in HepG2 cells treated with or without EGCG (Fig. 4a). PCSK9 has decreased accessibility in this assay (Fig. 4b). From the differential motif results, we found that two transcription factors related to PCSK9 binding site, HNF1α and FoxO3a were enriched in different groups-specific open regions (HNF1α motif was enriched in control, but FoxO3 motif was significantly enriched in EGCG treatment group) respectively, so we focused on these two TFs for further research (Fig. 4c).

### EGCG suppresses PCSK9 and promotes LDL uptake via activating FoxO3a but blocking HNF1α in cells

**Fig. 5:**
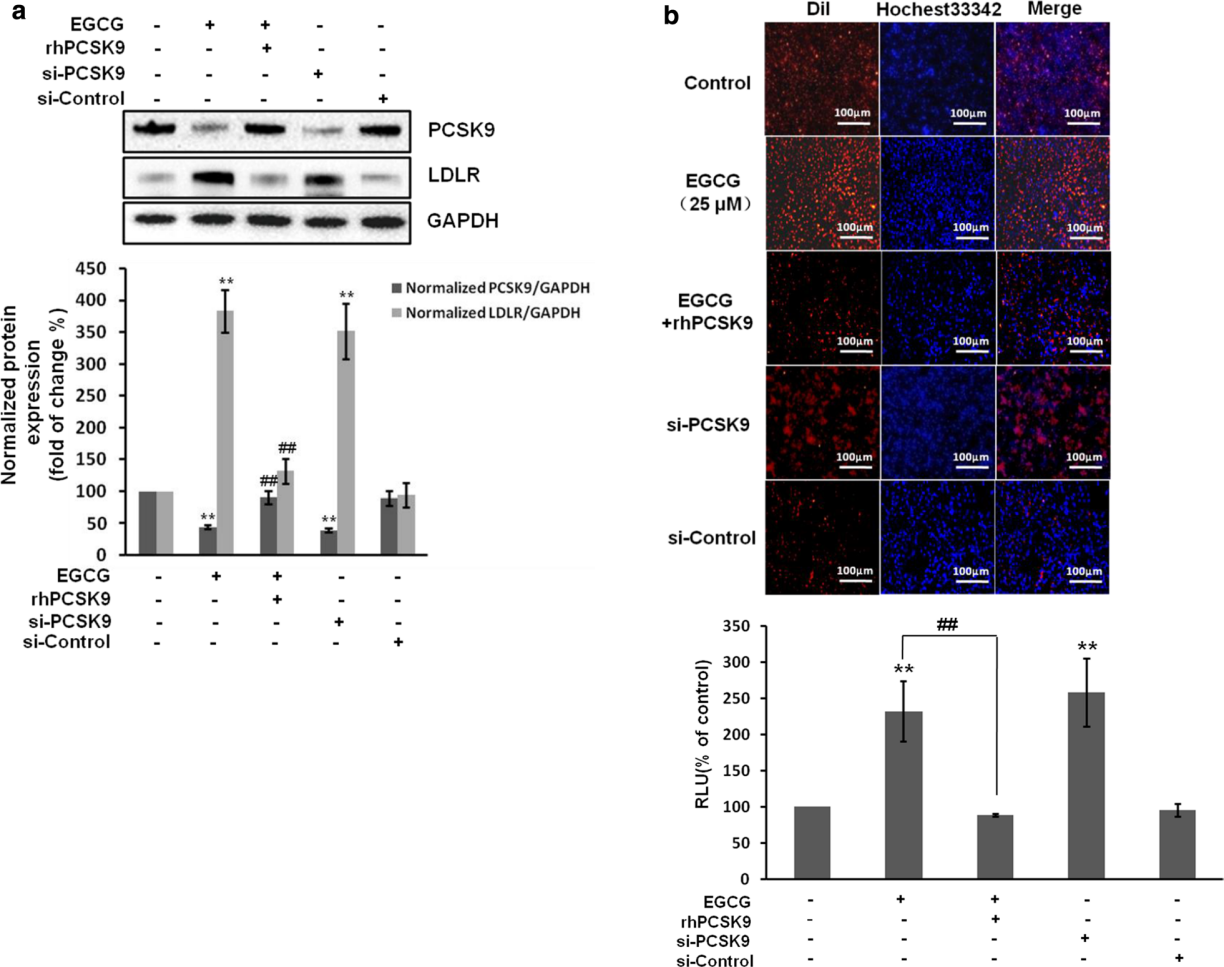
EGCG regulates LDLR expression and LDL uptake through PCSK9 in HepG2 cells. HepG2 cells were added rhPCSK9 or transfected with siRNA negative control (si-Control) or stealth siRNA for the knockdown of PCSK9 (si-PCSK9). **a** The levels of PCSK9 and LDLR were determined by western blot, GAPDH was used as the loading control. **b** Representative fluorescence microscopy images of cell‐associated Dil‐LDL (red), Hoechst‐stained nuclei (blue) and the overlay (upper). Fluorescence of isopropanol‐extracted Dil (520–570 nm, normalized to the cell protein) (lower). Data are presented as mean ± SEM (N = 3). **p < 0.01 compared with control. ##p < 0.01 compared with EGCG group. rhPCSK9: recombinant human

To verity that EGCG can increase LDL expression and LDL uptake by blocking PCSK9, cells were added recombinant human PCSK9 (rhPCSK9) or transfected with PCSK9 stealth siRNA. The results showed that rhPCSK9 was added can block the expression of LDLR and LDL uptake induced by EGCG, while transfection of PCSK9 stealth siRNA increased LDLR expression and LDL uptake (Fig. 5a, b). These results were confirmed in the Huh7 cells (Additional file 1: Figure S2A).

**Fig. 6:**
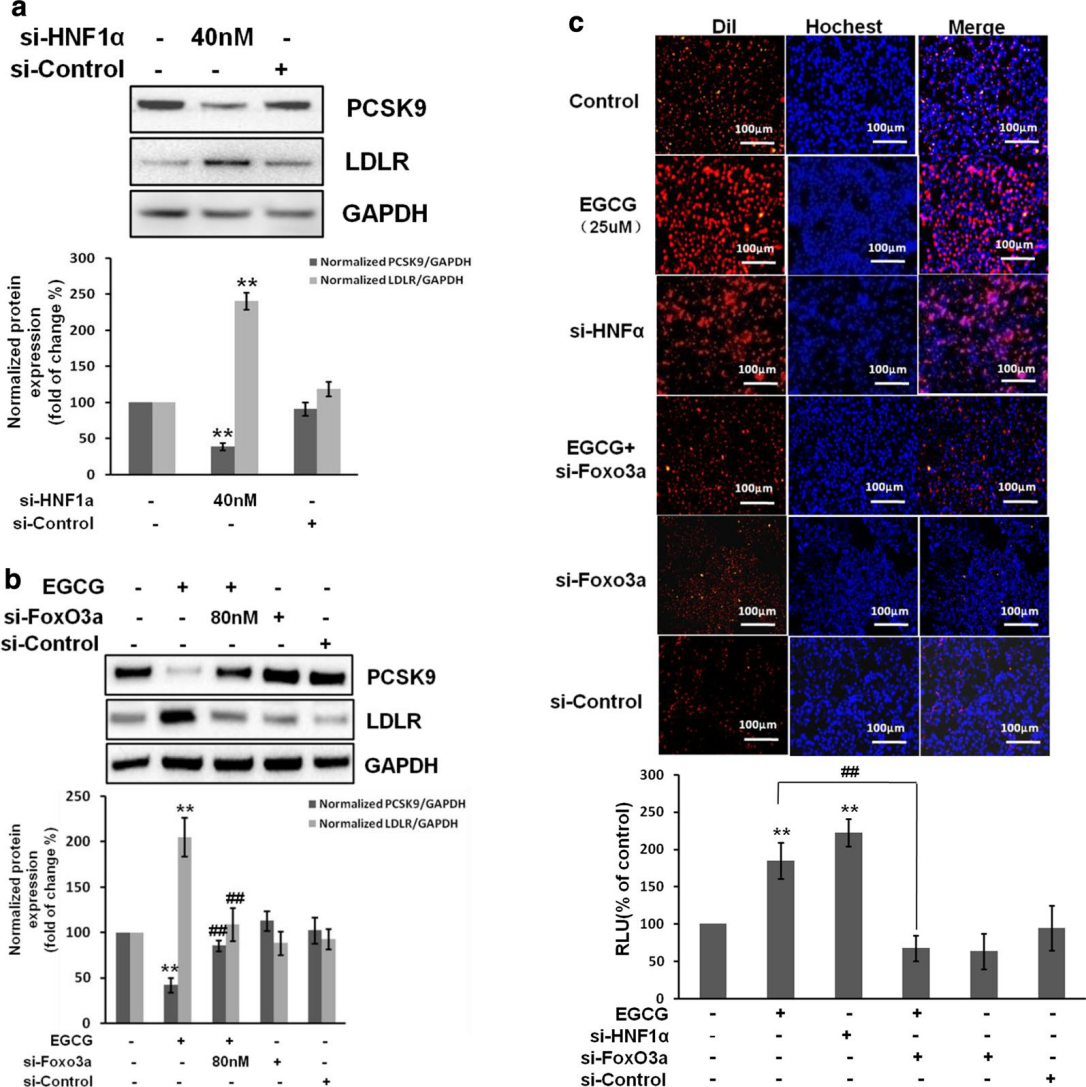
EGCG reduces PCSK9 but increases LDLR expression and activity were associated with FoxO3a and HNF1α in HepG2 cells. The nuclear extracts from with or without EGCG (25 μM) –treated HepG2 cells. **a** The effects of EGCG on the nuclear HNF1α and FoxO3a expression by Western blot analysis, the normalized intensity of nuclear HNF1α and FoxO3a protein, HDAC2 was used as the loading control. **b** HepG2 cells was transfected with siRNA negative control (si-Control) or stealth siRNA for the knockdown of HNF1α or FoxO3a (si-FoxO3a), the level of PCSK9 was determined by western blot, GAPDH was used as the loading control. **c** Representative fluorescence microscopy images of cell‐associated Dil‐LDL (red), Hoechst‐stained nuclei (blue) and the overlay (upper). Fluorescence of isopropanol‐extracted Dil (520–570 nm, normalized to the cell protein) (lower). Data are presented as mean ± SEM (N = 3). *p < 0.05, **p < 0.01 compared with control. ##p < 0.01 compared with EGCG group

To further explore the molecular mechanism of EGCG-mediated PCSK9 inhibiting in HepG2 cells, the nuclear proteins were extracted and the nuclear transcriptional regulators, including HNF1α and FoxO3a were detected by western blot analysis. Our data showed that the nuclear HNF1α expression was decreased approximately by 52.5 ± 5.9% and in EGCG (25 μM) treated cells. However, the nuclear FoxO3a expression was increased approximately by 60.7 ± 3.8%in EGCG-treated cells (Fig. 6a). The similar results were found in Huh7 cells (Additional file 1: Figure S2B, S2C).

To confirm that EGCG-reduced HNF1α is associated with PCSK9 reduction, cells transfected with HNF1α stealth siRNA alone for HNF1α knockdown, the level of PCSK9 was significantly decreased compared with control. Moreover, to demonstrate that EGCG-induced FoxO3a is associated with PCSK9 suppression, cells transfected with FoxO3a stealth siRNA, the expression of PCSK9 was significantly increased versus EGCG group. However, the LDLR level was increased by HNF1α stealth siRNA trasfection but decreased by FoxO3a stealth siRNA transfection (Fig. 6b). Besides, the LDL uptake activity induced by EGCG was blocked by FoxO3a stealth siRNA transfection. However, the LDL uptake was activated by HNF1α stealth siRNA transfection only (Fig. 6c). These experiments were performed in Huh7 cells and the similar results were obtained (Additional file 1: Figure S2D, S2E).

## Discussion

It has been demonstrated that frequent consumption of green tea has a great beneficial for lipid and cardiovascular system [26–28], which its underlying mechanism has not full been determined. In the present study performed in human, animal and in vitro. At first, the data for human clinical study showed that a lower level of LDL-C in the frequent tea consumption drinkers versus non-drinker controls, which is in agreement with previous clinical reports [19, 29]. Interestingly, the frequent tea consumption drinkers also had a reduction of PCSK9 level, a novel target for lipid metabolism. Furthermore, our animal and in vitro study further indicated that EGCG, the major content of green tea could significant increased LDLR, subsequently, enhanced LDL uptake, resulting in decreasing plasma LDL-C concentrations, which support the clinical finding. More important, EGCG exerted its effects on the lipid via PCSK9 inhibition and substantially involving in modulation of HNF1α and FoxO3a TFs. Such novel findings might provide the additional pivotal information in regard with the relation of EGCG and lipid.

As we well known, hyperlipidemia, especially abnormal elevation of plasma LDL-C levels, plays a key role in the pathogenesis of atherosclerotic CVD [30]. Therefore, lowering LDL-C constitutes the main approach to prevent and treat CVD, which can be achieved by reducing the synthesis of LDL-C and improving the clearance rate of LDL-C.

At present, there are nine members in PC family, namely PC1/3, PC2, furin, PC4, PC5/6, PACE4, PC7, SKI-1/S1P, and PCSK9. Some of these PCs play pivotal roles in regulating lipids and/or sterols. SKI-1/S1P regulates the synthesis of cholesterol and fatty acids. PC5A, PACE4, and/or furin cleave/inactivate EL and LPL, which are critical in HDL, VLDL, and chylomicron metabolism, PCSK9 enhances the degradation of the LDL receptor (LDLR) [31]. In addition, some of these PCs play essential role not only in neurodegeneration and tumor metastasis but also in regulating hormones [32–34]. Over the past decade, experimental and clinical studies have established that the key role of PCSK9 in the metabolism of LDL and LDLR as well as the verified safety of PCSK9 inhibition led to development of PCSK9 inhibitor. However, recent studies showed that nutraceuticals could provide a safe and cost-effective choice for inhibiting PCSK9 [18, 35]. For example, quercetin is a well-documented antioxidant flavonoid present in a wide range of vegetables and fruits. Several experimental studies have shown that quercetin effectively reduces AS plaque and lipid deposition, which may be related to regulation of ABCA1, LXR and PCSK9 expression in mice [7, 36]. Curcumin is a polyphenolic compound which is extracted from the rhizomes of turmeric. Tai et al. [37] investigated that curcumin can improve LDL-C uptake through inhibition of PCSK9 and upregulation of LDLR expression on the surface of HepG2 cells. Our results suggested that EGCG, a natural tea polyphenol may play a potential role in suppressing PCSK9.

Over the past decades, the traditional conception that green tea consumption offers a sense of beneficial effects for human health, particularly in the areas of cardiovascular diseases [38, 39]. Nicolas Danchin, a key note speaker reported that drinking tea can lower the risk of non-cardiovascular mortality by 24% at 2014 ESC Congress. In the present study, we found that the circulating PCSK9 levels of regular green tea drinkers were lower than those of non-tea drinkers, even reduced the LDL-C concentrations. However, EGCG is one of the major active tea polyphenol in green tea. Some experiments revealed that EGCG can exert a variety of physiological actions, including antioxidant, anti-inflammatory, anti-hypertensive, and anti-diabetic effects [18]. Several recent results suggested that EGCG has been shown to reduce the development of atherosclerosis and the progression of evolving atherosclerotic lesions in mice [40, 41]. Another study demonstrated that ECGC improves hypercholesterolemia by interfering with the absorption of dietary cholesterol [42]. In human, we found that the circulating PCSK9 levels of regular green tea drinkers were lower than those of non-tea drinkers, even reduced the LDL-C concentrations. In animal, we also found that EGCG decreased both hepatic and circulating PCSK9 levels but promoted hepatic LDLR expression, and then reduced blood LDL-C in HFD rats. Recently, Kitamura et al. [20] demonstrated that EGCG promotes LDLR but inhibits PCSK9 levels through Annexin A2 pathway in HepG2 cells. However, we used the ATAC-seq technology and found that other factors may be involved in EGCG regulation of LDL uptake through PCSK9 pathway.

Studies have shown that many transcription factors are involved in the regulation of PCSK9. HNF1α has been previously identified as the predominant trans-activator for PCSK9 [43, 44]. Berberine inhibited the expression of PCSK9 through decreasing HNF1α and sterol regulatory element binding protein-2 (SREBP2) [45–47]. Tai et al. [37] reported that curcumin reduced PCSK9 via down regulating HNF1α. FoxO TFs have been reported to play an important role in several essential cellular functions including cell proliferation, apoptosis, lipid metabolism and liver function [25, 48, 49]. Among these FoxO proteins, FoxO3a is a predominant player in the regulation of cholesterol homeostasis in hepatic cells. It has been reported that the loss of FoxO3a leads to increased lipid synthesis in the liver [25, 50]. In hepatic FoxO3-deficient mice, both the hepatic and plasma cholesterol levels were increased. However, the overexpression of FoxO3a improves hypercholesterolemia in obese mouse models [51]. Chen et al. [52] revealed that tanshinone IIA can inhibit the expression of PCSK9 through increasing the level of FoxO3a in HepG2 cells. In this study, we use assay for (ATAC-seq) [24] to show that EGCG regulates chromatic accessibility open region and find some potential TFs in HepG2 cells. But it is not clear whether two TFs, HNF-1α and FoxO3 are involved in PCSK9 regulation by EGCG. We focused on these two TFs for further study. Our data suggested that EGCG increased LDLR expression and thereby leading to enhanced LDL uptake activity by decreasing PCSK9 via both activation of FoxO3a and blocking HNF1α in HepG2 cells. The similar results were confirmed in Huh7 cells.

In addition, we analyzed regarding the comparison of clinical characteristics between those who frequently or never drink tea. There was no significant difference regarding BMI, DM, hypertension, CAD, smoke, drink, HDL-C and TG between two groups. Moreover, multivariate linear regression analysis also showed that tea consumption was independently correlated with plasma PCSK9 level (Additional file 1: Tables S1 and S2). However, a study on large sample size with prospective manner may be needed to confirm our findings.

## Conclusion

**Fig. 7:**
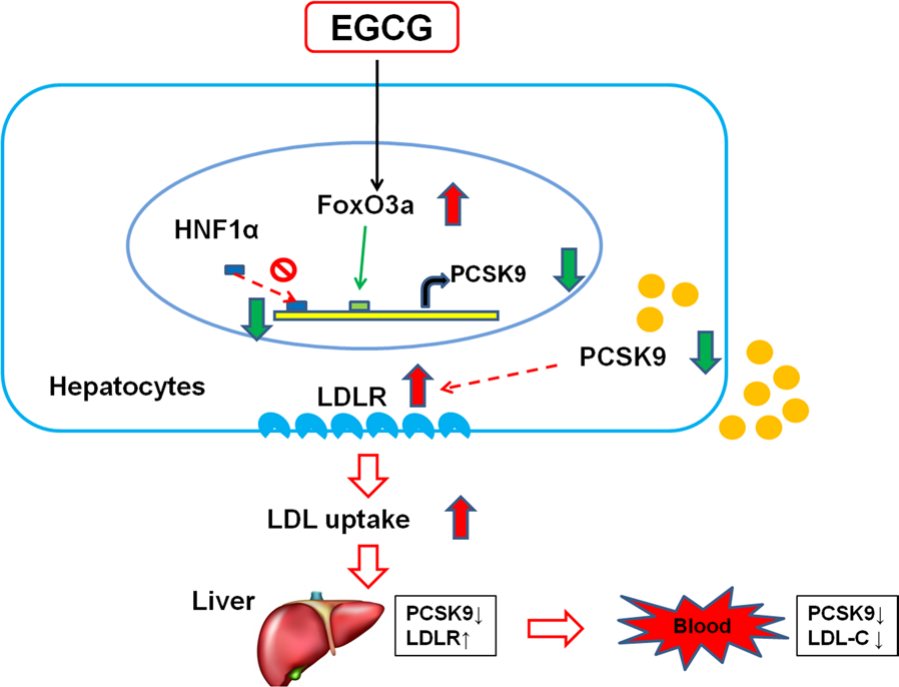
Schematic summary of hypothetic mechanism of EGCG regulates LDL-C through inhibiting PCSK9. EGCG suppresses PCSK9 production by promoting nuclear FoxO3a, and reducing nuclear HNF1α, resulting in up-regulated LDLR expression and LDL uptake in hepatocytes. Thereby inhibiting liver and circulating PCSK9 levels, and ultimately lowering LDL-C levels

In summary, the current study provides experimental basis on green tea or EGCG as a potential, natural PCSK9 inhibitor regulates cholesterol. Our findings indicated that EGCG suppresses PCSK9 production by promoting nuclear FoxO3a, and reducing nuclear HNF1α,resulting in up-regulated LDLR expression and LDL uptake in hepatocytes. Thereby inhibiting liver and circulating PCSK9 levels, and ultimately lowering LDL-C levels (Fig. 7). Such findings may have important clinical implication due to the features of green tea, such as easily available, relatively cheap, and favorable taste. More studies may be needed to confirm our findings in the future.

## Supplementary information

**Additional file 1: Figure S1.** The effects of EGCG on circulating PCSK9 level in dose-dependent manner in rats. SD rats fed with HFD for 4 weeks and administered with EGCG by i.g. at different doses for another 4 weeks. The levels of plasma PCSK9 were analyzed by ELISA kit. **p <0.01, vs control, #p <0.05, ##p <0.01, versus HFD group (N=3). **Figure S2.** EGCG modulates LDLR and LDL uptake through PCSK9-dependent pathway by regulating HNF1α and FoxO3a in hepatic cells. (A) Cells were added rhPCSK9 or transfected with siRNA negative control (si-Control) or stealth siRNA for the knockdown of PCSK9 (si-PCSK9). The levels of PCSK9 and LDLR were determined by western blot, GAPDH was used as the loading control. (B) The nuclear extracts from with or without EGCG (25 μM) –treated Huh7 cells. The effects of EGCG on the nuclear HNF1α and FoxO3a expression by western blot analysis, the normalized intensity of nuclear HNF1α and FoxO3a protein, HDAC2 was used as the loading control. (C) Cells were transfected with siRNA negative control (si-Control) or stealth siRNA for the knockdown of HNF1α, the level of PCSK9 and LDLR were determined by western blot, GAPDH was used as the loading control. (D) Cells were transfected with siRNA negative control (si-Control) or stealth siRNA for the knockdown of FoxO3a, the level of PCSK9 and LDLR were determined by western blot, GAPDH was used as the loading control. (E) Representative fluorescence microscopy images of cell‐associated Dil-LDL (red), Hoechst‐stained nuclei (blue) and the overlay (upper). Fluorescence of isopropanol‐extracted Dil (520-570 nm, normalized to the cell protein) (lower). Data are presented as mean±SEM (N=3). **p <0.01 compared with control. ##p <0.01 compared with EGCG group. **Figure S3.** Flow charts of the experiment. We studied the effects and underlying molecular mechanism of EGCG or green tea on regulating cholesterol from human, animal and *in vitro,* this chart shows the whole experiment process. **Table S1.** Baseline characters of study population. **Table S2.** Univariate and multivariate linear regression analyses for the relationship of PCSK9 and tea consumption.

## Data Availability

The relevant raw data will not be shared because the data from cell described and presented in the manuscript are completed and clear for testing by reviewers.

## Abbreviations

ATAC-seq: Assay for transposase-accessible chromatic with high-throughput sequencing
CVD: Cardiovascular disease
EGCG: Epigallocatechingallate
HepG2: Human hepatocellular carcinoma cell line 2
FoxO: Forkhead box class O
GAPDH: Glyceraldehyde-3-phosphate dehydrogenase
HFD: High fat diet
HNF-1α: Hepatocyte nuclear factor-1α
i.g.: Intragastric gavage
LDL-C: Low-density lipoprotein cholesterol
LDLR: Low-density lipoprotein receptor
LPDS: Lipoprotein-deficient serum
ND: Normal chow diet
PCSK9: Proprotein convertase subtilisin/kexin type 9
SD: Sprague-Dawly
TF: Transcription factor
