# Supplementary material for: Beneficial impact of epigallocatechingallate on LDL-C through PCSK9/LDLR pathway by blocking HNF1α and activating FoxO3a
Source: J Transl Med. 2020 May 12;18:195. doi: 10.1186/s12967-020-02362-4 (PMC7216725; doi:10.1186/s12967-020-02362-4)
Supplement: Supplementary file 1 — Additional file 1: Figure S1. The effects of EGCG on circulating PCSK9 level in dose-dependent manner in rats. SD rats fed with HFD for 4 weeks and administered with EGCG by i.g. at different doses for another 4 weeks. The levels of plasma PCSK9 were analyzed by ELISA kit. **p <0.01, vs control, #p <0.05, ##p <0.01, versus HFD group (N=3). Figure S2. EGCG modulates LDLR and LDL uptake through PCSK9-dependent pathway by regulating HNF1α and FoxO3a in hepatic cells. (A) Cells were added rhPCSK9 or transfected with siRNA negative control (si-Control) or stealth siRNA for the knockdown of PCSK9 (si-PCSK9). The levels of PCSK9 and LDLR were determined by western blot, GAPDH was used as the loading control. (B) The nuclear extracts from with or without EGCG (25 μM) –treated Huh7 cells. The effects of EGCG on the nuclear HNF1α and FoxO3a expression by western blot analysis, the normalized intensity of nuclear HNF1α and FoxO3a protein, HDAC2 was used as the loading control. (C) Cells were transfected with siRNA negative control (si-Control) or stealth siRNA for the knockdown of HNF1α, the level of PCSK9 and LDLR were determined by western blot, GAPDH was used as the loading control. (D) Cells were transfected with siRNA negative control (si-Control) or stealth siRNA for the knockdown of FoxO3a, the level of PCSK9 and LDLR were determined by western blot, GAPDH was used as the loading control. (E) Representative fluorescence microscopy images of cell‐associated Dil-LDL (red), Hoechst‐stained nuclei (blue) and the overlay (upper). Fluorescence of isopropanol‐extracted Dil (520-570 nm, normalized to the cell protein) (lower). Data are presented as mean±SEM (N=3). **p <0.01 compared with control. ##p <0.01 compared with EGCG group. Figure S3. Flow charts of the experiment. We studied the effects and underlying molecular mechanism of EGCG or green tea on regulating cholesterol from human, animal and in vitro, this chart shows the whole experiment process. Table S1. Baseline char [file 12967_2020_2362_MOESM1_ESM.pdf]

Supplementary figures and legends

Figure S1. The effects of EGCG on circulating PCSK9 level in dose-dependent manner in rats. SD rats fed with HFD for 4 weeks and administered with EGCG by i.g. at different doses for another 4 weeks. The levels of plasma PCSK9 were analyzed by ELISA kit. **p <0.01, vs control, $^{\#}$p <0.05, $^{\#\#}$p <0.01, versus HFD group (N=3).

Figure S2. EGCG modulates LDLR and LDL uptake through PCSK9-dependent pathway by regulating HNF1α and FoxO3a in Huh7 cells. (A) Cells were added rhPCSK9 or transfected with siRNA negative control (si-Control) or stealth siRNA for the knockdown of PCSK9 (si-PCSK9). The levels of PCSK9 and LDLR were determined by western blot, GAPDH was used as the loading control. (B) The nuclear extracts from with or without EGCG (25 μM) –treated Huh7 cells. The effects of EGCG on the nuclear HNF1α and FoxO3a expression by western blot analysis, the normalized intensity of nuclear HNF1α and FoxO3a protein, HDAC2 was used as the loading control. (C) Cells were transfected with siRNA negative control (si-Control) or stealth siRNA for the knockdown of HNF1α, the level of PCSK9 and LDLR were determined by western blot, GAPDH was used as the loading control. (D) Cells were transfected with siRNA negative control (si-Control) or stealth siRNA for the knockdown of FoxO3a, the level of PCSK9 and LDLR were determined by western blot, GAPDH was used as the loading control. (E) Representative fluorescence microscopy images of cell‐associated DiI-LDL (red), Hoechst‐stained nuclei (blue) and the overlay (upper). Fluorescence of isopropanol‐extracted DiI (520-570 nm, normalized to the cell protein) (lower). Data are presented as mean±SEM (N=3). **p <0.01 compared with control. $^{\#\#}$p <0.01 compared with EGCG group.

Figure S3. Flow charts of the experiment. We studied the effects and underlying molecular mechanism of EGCG or green tea on regulating cholesterol from human, animal and *in vitro,* this chart shows the whole experiment process.

Figure S1.

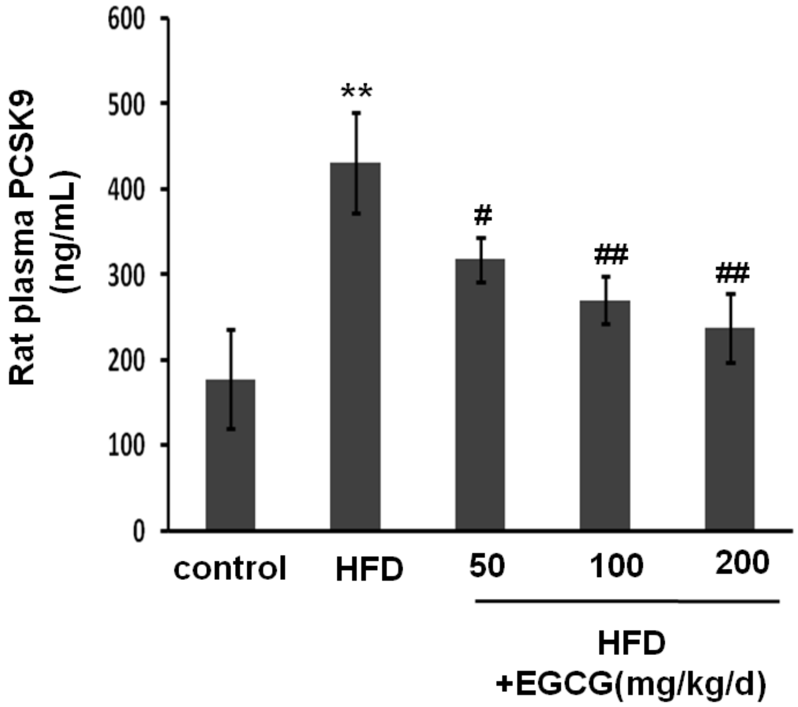

Rat plasma PCSK9 (ng/mL)
600
500
400
300
200
100
0
**
#
##
##
control
HFD
50
100
200
HFD
+EGCG(mg/kg/d)

Figure S2.

A.

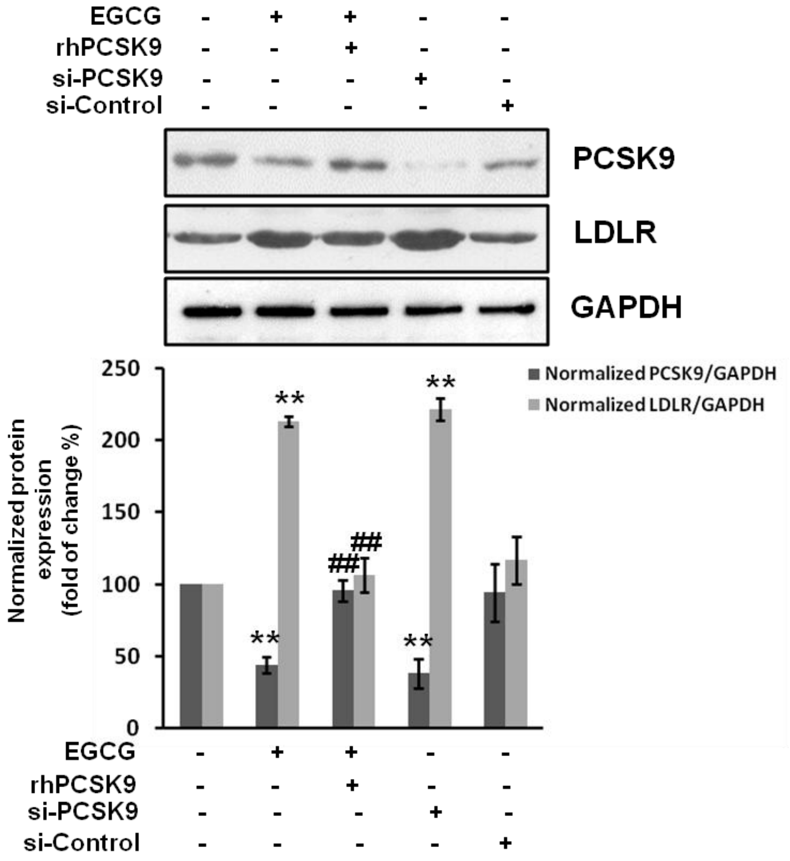
EGCG - + + - -
rhPCSK9 - - + - -
si-PCSK9 - - - + -
si-Control - - - - +
PCSK9
LDLR
GAPDH
Normalized protein expression (fold of change %)
Normalized PCSK9/GAPDH
Normalized LDLR/GAPDH
250
200
150
100
50
0
**
**
##
##
**
**
EGCG - + + - -
rhPCSK9 - - + - -
si-PCSK9 - - - + -
si-Control - - - - +

B.

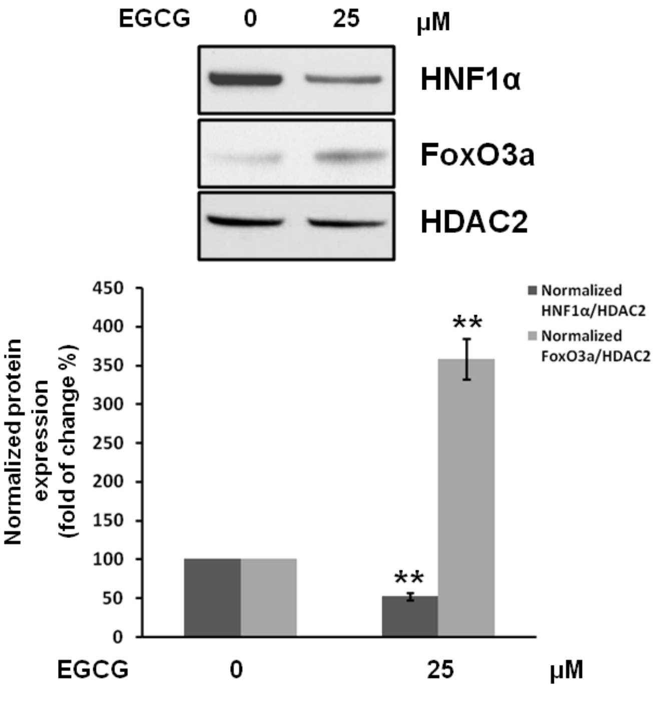
EGCG 0 25 μM
HNF1α
FoxO3a
HDAC2
Normalized protein expression (fold of change %)
Normalized HNF1α/HDAC2
Normalized FoxO3a/HDAC2
450
400
350
300
250
200
150
100
50
0
**
**
EGCG 0 25 μM

C.

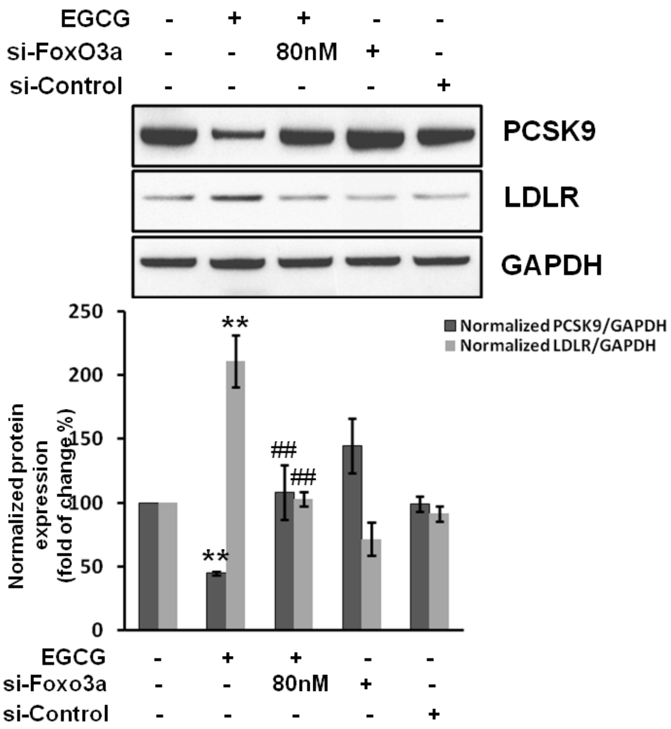

EGCG - + + - -
si-FoxO3a - - 80nM + -
si-Control - - - - +
PCSK9
LDLR
GAPDH
Normalized PCSK9/GAPDH
Normalized LDLR/GAPDH
Normalized protein expression (fold of change %)
250
200
150
100
50
0
**
**
##
##
EGCG - + + - -
si-Foxo3a - - 80nM + -
si-Control - - - - +

D.

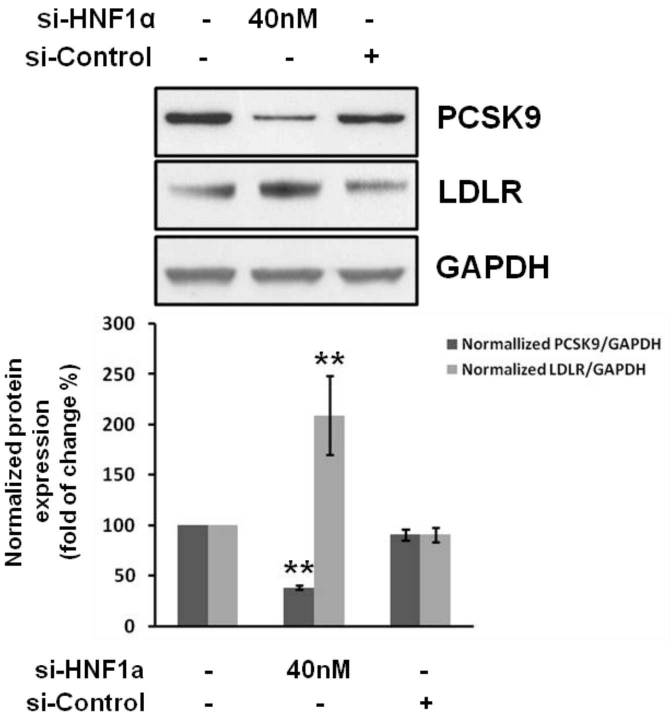

si-HNF1α - 40nM -
si-Control - - +
PCSK9
LDLR
GAPDH
Normallized PCSK9/GAPDH
Normalized LDLR/GAPDH
Normalized protein expression (fold of change %)
300
250
200
150
100
50
0
**
**
si-HNF1a - 40nM -
si-Control - - +

E.

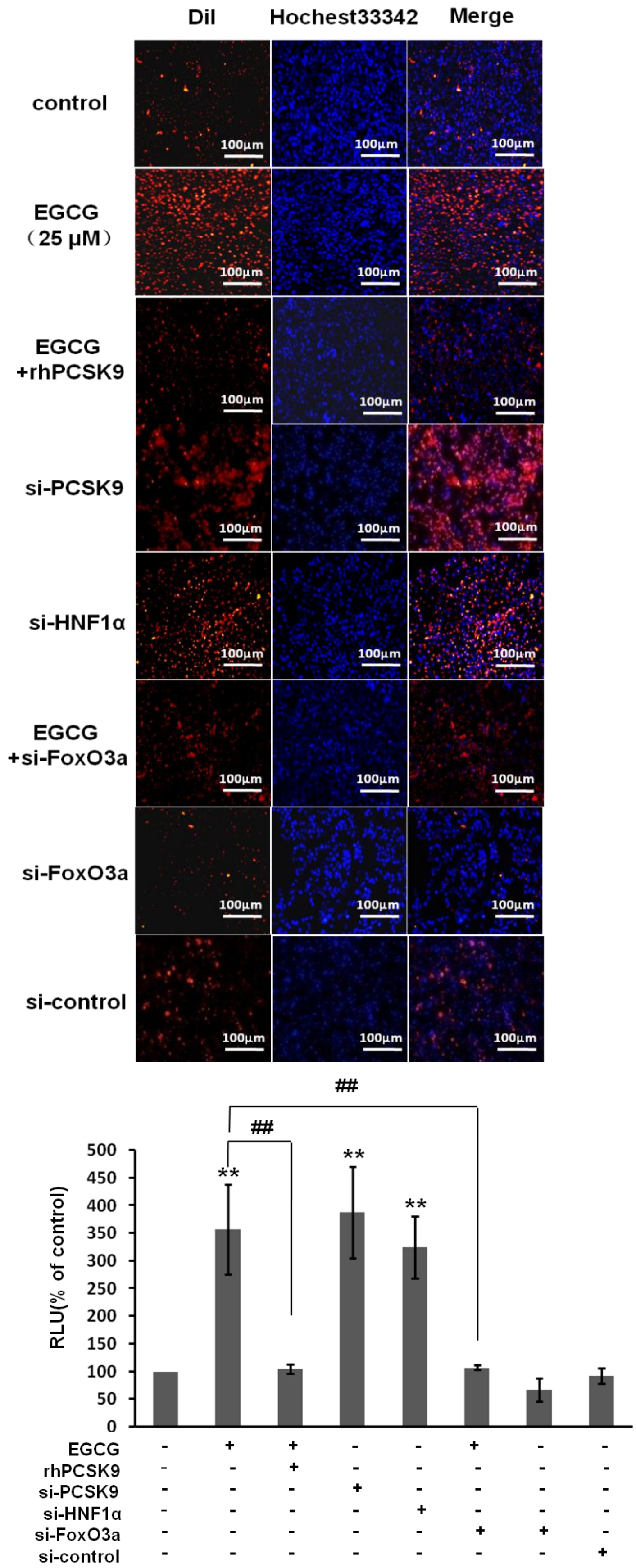

Dil
Hochest33342
Merge
control
EGCG
（25 μM）
EGCG
+rhPCSK9
si-PCSK9
si-HNF1α
EGCG
+si-FoxO3a
si-FoxO3a
si-control
100μm
##
##
**
**
**
RLU(% of control)
500
450
400
350
300
250
200
150
100
50
0
EGCG
rhPCSK9
si-PCSK9
si-HNF1α
si-FoxO3a
si-control

Figure S3.

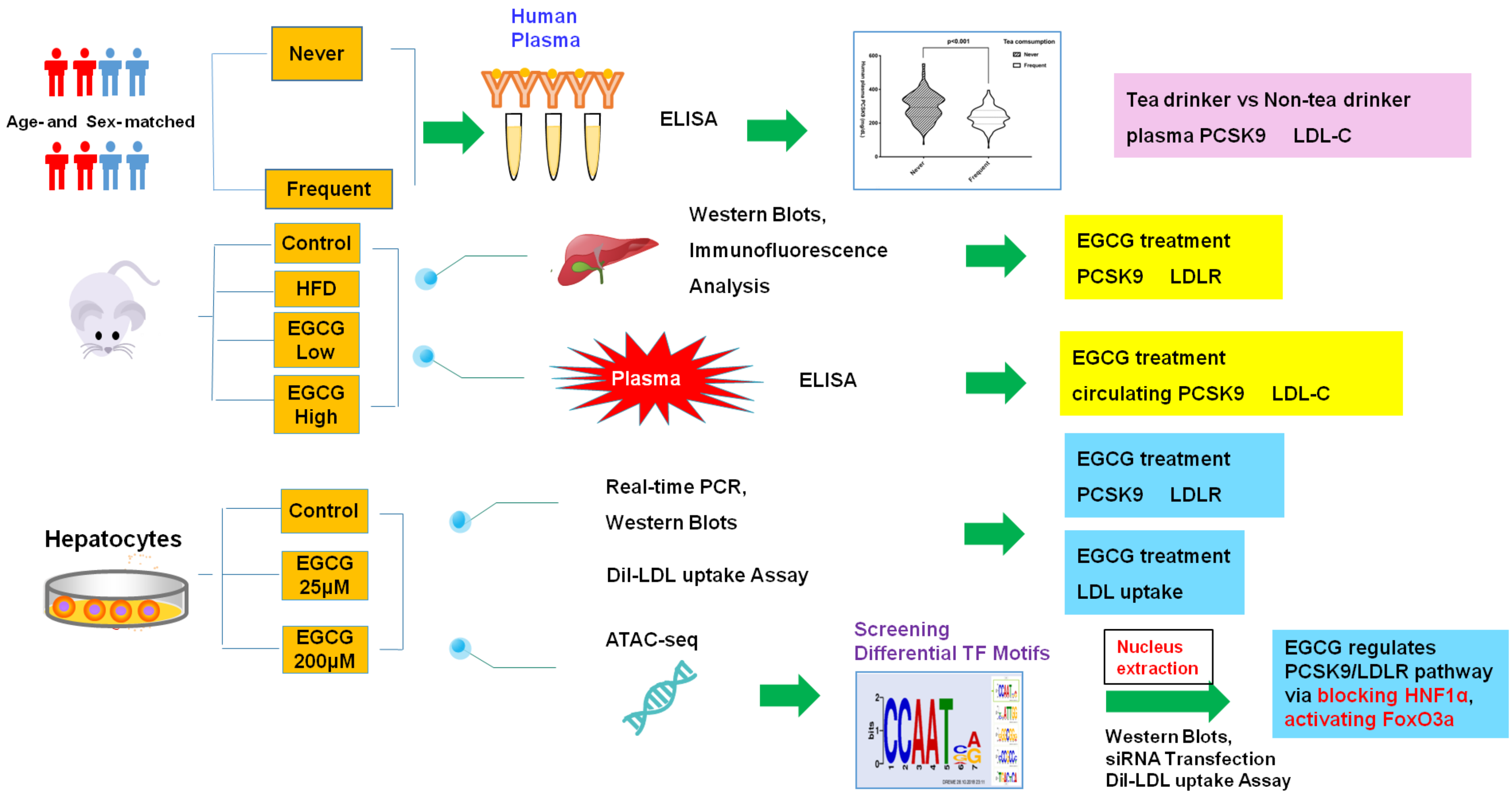
Age- and Sex-matched
Never
Frequent
Human Plasma
ELISA
Tea drinker vs Non-tea drinker
plasma PCSK9 LDL-C
Control
HFD
EGCG Low
EGCG High
Western Blots,
Immunofluorescence
Analysis
EGCG treatment
PCSK9 LDLR
Plasma
ELISA
EGCG treatment
circulating PCSK9 LDL-C
Hepatocytes
Control
EGCG 25μM
EGCG 200μM
Real-time PCR,
Western Blots
DiI-LDL uptake Assay
EGCG treatment
PCSK9 LDLR
EGCG treatment
LDL uptake
ATAC-seq
Screening Differential TF Motifs
Nucleus extraction
Western Blots,
siRNA Transfection
DiI-LDL uptake Assay
EGCG regulates PCSK9/LDLR pathway via blocking HNF1α, activating FoxO3a

Table S1. Baseline characters of study population

	Tea consumption		
**Variables**	**Never, n=224**	**Frequent, n=224**	**P**
**Age, years**	53.7±10.5	54.3±10.6	-
**Male, n (%)**	155(69.2)	155(69.2)	-
**BMI, kg/m^2**	25.6±3.3	25.4±3.4	0.813
**DM, n (%)**	88(39.3)	80(35.7)	0.435
**Hypertension**	121(54.0)	110(49.1)	0.298
**CAD, n (%)**	123(55.8)	116(51.8)	0.394
**Smoke, n (%)**	95(42.4)	107(47.8)	0.255
**Drink, n (%)**	47(21.0)	53(23.7)	0.496
**LDL-C (mmol/L)**	3.05±0.92	3.26±1.02	0.025
**HDL-C (mmol/L)**	1.13±0.34	1.12±0.42	0.734
**TG (mmol/L)**	1.72(1.23-2.26)	1.70(1.14-2.50)	0.739
**PCSK9 (mg/dL)**	236.8±57.7	297.4±81.6	<0.001

Data were expressed as mean ± SD, median with 25th and 75th percentile or n (%).

BMI: body mass index; DM: diabete mellitus; CAD: coronary artery disease; TG: triglyceride; LDL-C: low density lipoprotein cholesterol; HDL-C: high density lipoprotein cholesterol.

Table S2. Univariate and multivariate linear regression analyses for the relationship of PCSK9 and tea consumption

Variables	Univariate regression		Multivariate regression	
	Standard Coefficients	P	Standard Coefficients	P
**BMI**	0.075	0.146	0.043	0.382
**Hypertension**	0.090	0.057	0.025	0.617
**DM**	0.050	0.295	-0.013	0.778
**Smoke**	-0.006	0.900	0.012	0.810
**Drink**	-0.018	0.705	-0.032	0.527
**CAD**	0.163	0.001	0.114	0.026
**TG**	-0.022	0.643	0.030	0.527
**HDL-C**	0.100	0.034	0.046	0.350
**LDL-C**	0.271	<0.001	0.136	0.008
**Tea consumption**	-0.362	<0.001	-0.330	<0.001
